# Implementation of Remote Patient Monitoring and Earlier CERT Activation: Effects on ICU Transfer and Mortality

**DOI:** 10.3390/jcm14207434

**Published:** 2025-10-21

**Authors:** Victor Narcisse, Farhan Ishaq, Melissa Gomez, Sarah Homer, Laura Griffin, Sarah Pletcher, Ngoc-Anh Nguyen

**Affiliations:** 1 Department of Medicine, Houston Methodist Hospital, Houston, TX 77030, USA; mlkuerbitz@houstonmethodist.org (M.G.); shomer@houstonmethodist.org (S.H.); lmgriffin@houstonmethodist.org (L.G.); nanguyen@houstonmethodist.org (N.-A.N.); 2 Houston Methodist Research Institute, Houston Methodist Hospital, Houston, TX 77030, USA; spletcher@houstonmethodist.org; 3 Center for Connected Care, Innovation & Implementation Research, Houston Methodist Academic Institute, Houston Methodist Hospital, Houston, TX 77030, USA; 4 Department of Surgery, Houston Methodist Hospital, Houston, TX 77030, USA

**Keywords:** BioButton, remote patient monitoring, clinical emergency response team, rapid response team

## Abstract

**Introduction:** Timely detection of clinical deterioration in hospitalized patients remains a challenge, often limited by intermittent vital signs (VS) monitoring and delayed escalation. Remote patient monitoring (RPM) offers a medium of high-frequency surveillance of patient VS and may facilitate earlier recognition of deterioration. This study evaluated whether RPM integration into rapid response workflows improves clinical outcomes among patients requiring clinical emergency response team (CERT) activation and subsequent intensive care unit (ICU) transfer. **Methods:** A retrospective study was conducted to assess the impact of RPM implementation on severity of illness and mortality in adult patients who experienced CERT activation followed by ICU transfer. The primary outcomes were severity of illness at ICU admission and in-hospital mortality. We hypothesized that patients in the post-intervention group would demonstrate better outcomes compared to pre-intervention. **Results:** A total of 1120 patients were included (PRE: n = 656; POST: n = 464). The POST group, which received continuous monitoring via the BioButton^®^ device and augmented workflows, demonstrated a lower mean APACHE-IV score at ICU transfer (83.96 vs. 90.01; *p* = 0.0016 and reduced in-hospital mortality (7.75% vs. 11.48%; *p* = 0.084). Median ICU stay in the PRE group was 5.85 (3.00–11.58) and 5.07 (2.59–9.22) in the POST group (*p*: 0.0565). Total LOS was 11.95 (6.57–20.40) and 10.50 (6.01–18.17), respectively [*p* = 0.0278]. **Conclusions:** Integration of RPM into hospital care pathways was associated with earlier recognition of clinical deterioration, reduced illness severity at ICU admission, and lower in-hospital mortality. These findings may support the utility of RPM as part of a comprehensive, multicomponent, rapid response model to recognize early physiological deterioration and may improve patient safety and outcomes in acute care settings.

## 1. Introduction

Timely recognition of clinical deterioration in hospitalized patients remains a persistent challenge in acute care medicine. Despite the widespread use of early warning scores (e.g., MEWS and NEWS) [1,2,3], clinician-driven assessments, and rapid response systems (RRS) [4,5], many adverse events continue to go undetected until patients require escalation to intensive care [6]. A key limitation of these traditional strategies is their reliance on intermittent vital signs (VS) monitoring, which may miss subtle but clinically significant trends [7,8]. Early warning systems often lack the sensitivity and timeliness required for proactive intervention [9]. Additionally, as the U.S. population ages [10], hospitals are encountering more medically complex patients requiring closer monitoring, a factor increasing rapid response call volume [11].

In response to these challenges, remote patient monitoring (RPM) has emerged as a promising solution, offering high-frequency physiologic surveillance through wearable devices [12]. By capturing real-time metrics such as heart rate, respiratory rate, and skin temperature, RPM enables earlier detection of decompensation that may not be evident through periodic assessments alone. This continuous data stream opens opportunities to trigger rapid intervention and improve patient outcomes. This may be particularly beneficial at times when resources are more limited, for example, during night shifts [13].

## 2. Study Setting

At Houston Methodist Hospital (HMH), a tertiary academic medical center in Houston, Texas, a novel clinical deterioration response model has been implemented. This system integrates RPM into the workflows of the Clinical Emergency Response Team (CERT), virtual intensive care unit (vICU), and bedside clinical teams. CERT, staffed by nurse practitioners 24/7, provides rapid response services across inpatient units and observation areas. Although activations may be initiated by physicians or advanced practice providers, bedside nurses historically trigger the majority of events based on their direct clinical observations.

To enhance early detection capabilities, HMH deployed wearable RPM devices that collect and transmit continuous physiological data. These efforts consisted of a multicomponent initiative and were supported by clinical workflow redesign and the integration of algorithm-based monitoring and AI-assisted decision support tools. Evidence supports the clinical value of continuous vital sign monitoring, which has been associated with decreased ICU transfers and shorter hospital stays in medical–surgical units [12]. These outcomes reinforce the hypothesis that continuous surveillance enables earlier intervention and mitigates the severity of deterioration [12].

## 3. Objective

This study aims to evaluate whether the integration of RPM into acute care workflows improves outcomes for patients requiring CERT activation and subsequent ICU transfer. Specifically, we examine two primary endpoints: (1) the severity of illness at ICU admission, measured by the Acute Physiology and Chronic Health Evaluation IV (APACHE-IV) score, and (2) in-hospital mortality. Secondary endpoints included length of stay (LOS). We hypothesize that RPM facilitates earlier recognition of deterioration, thereby reducing illness severity at ICU transfer and improving survival. Additionally, the study explores whether operational enhancements—including artificial intelligence (AI)-based tools and clinical escalation algorithms—have contributed to the optimization of rapid response interventions.

The system evaluated in this study was the BioButton^®^ (BioIntelliSense, Inc., Golden, CO, USA), a wearable medical device approximately the size of a silver dollar that enables automated, high-frequency vital sign (VS) monitoring (Supplementary Materials). The device captures up to 1440 sets of measurements per day, continuously recording key physiologic parameters such as respiratory rate, heart rate, and skin temperature [7]. This continuous data stream facilitates earlier detection of clinical deterioration while significantly reducing the cost and labor burden associated with traditional manual vs. automated collection, which typically occurs only four to six times daily [14].

## 4. Methods

A retrospective cohort study was conducted at a 1020-bed tertiary academic hospital in the Texas Medical Center (Houston, TX, USA) to evaluate the impact of RPM on outcomes among patients who experienced clinical deterioration. The study population included adult patients admitted to medical–surgical units under inpatient or observation status who required CERT activation, followed from January 2022 through July 2024, by transfer to the ICU from acute care. The specific assignment of groups based on unit-specific rolling RPM go-live dates. CERT activations from 7:00 AM to 6:59 PM were classified as daytime CERT activations; nighttime CERT activations were from 7:00 PM to 6:59 AM.

### 4.1. Study Design and Population

**Figure 1:**
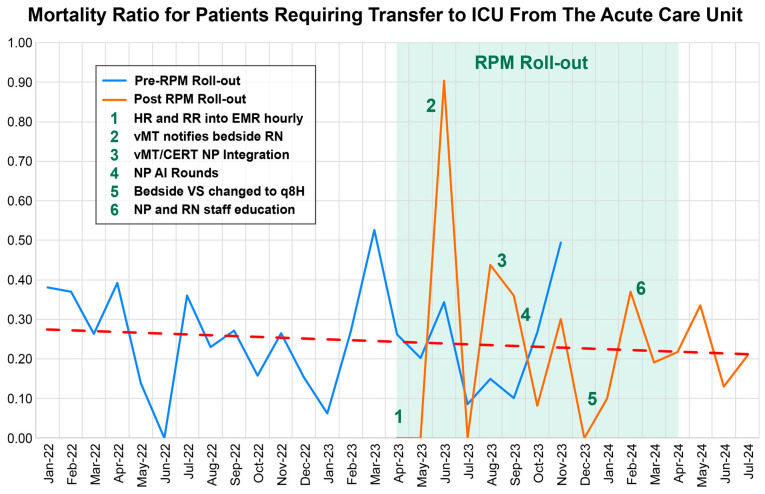
Monthly mortality ratio for patients requiring transfer to the ICU from acute care units between January 2022 and July 2024. The blue line represents the mortality during the pre–RPM period, while the orange line represents the mortality during the post-RPM period. The dashed red line represents the trend of mortality.

Patients were stratified into two cohorts based on the timeline of RPM implementation across nursing units; as implementation varied across units, there was an overlap between the pre- and post-intervention period (Figure 1).

***Pre-intervention group (Control)*:** January 2022 through October 2023, during which standard care protocols were followed. These included routine vs. collection every four hours and hourly clinical staff rounding.

***Post-intervention group (Intervention)*:** April 2023 to July 2024, during which RPM technologies and augmented workflows were introduced.

RPM was implemented in a rolling fashion across medical–surgical units, with each unit launching the intervention on a different date. As a result, the number of months contributing to pre- and post-intervention data varied across units. For analytic consistency, patients were categorized as pre- or post-intervention based on the specific date of RPM activation in their respective unit. While this resulted in unequal timeframes between groups overall, it ensured accurate classification relative to intervention exposure.

The primary objective was to determine whether high-frequency physiologic monitoring—enabled through wearable technology and electronic medical record (EMR)–based analytics—could facilitate earlier detection of deterioration and improve patient outcomes. Severity of illness at the time of ICU transfer was assessed using Acute Physiology and Chronic Health Evaluation IV (APACHE-IV) scores.

### 4.2. Intervention Components

A multi-component RPM strategy was deployed (Figure 1) during the post-intervention period, including:

RPM with BioButton^®^ (BioIntelliSense Inc., Golden, CO, USA): A wearable sensor that continuously measures respiratory rate, resting heart rate, and skin temperature.

Hot Spot Rounds: Conducted by CERT nurse practitioners using AI-driven patient risk stratification tools to proactively identify high-risk individuals.

High-Frequency vs. Charting: Hourly pulse and respiratory rate measurements integrated into EMR flowsheets to improve real-time data availability.

Virtual ICU (vICU) Support: Continuous physiologic data review by critical care clinicians with alerts communicated to bedside staff.

CERT Feedback Loop: Educational reinforcement through “good catch” stories that highlighted successful early interventions.

This high-risk patient population—those requiring ICU transfer following CERT activation—was intentionally selected to assess the effectiveness of high-frequency physiologic monitoring in individuals with substantial illness burden, thereby enriching the dataset with clinically significant deterioration events. This methodology reduced the confounding of CERT events initiated for potentially non-critical medical concerns.

The phased implementation ensured that pre- and post-intervention cohorts remained discrete, minimizing contamination; the technological requirements of the post-intervention group further reduced the risk of contamination. Units that had not yet initiated RPM continued standard care, while those that adopted RPM transitioned fully to the intervention protocol at the designated go-live date.

This project was reviewed and deemed exempt by the Institutional Review Board.

### 4.3. Statistical Analysis

All analyses were conducted using Stata (version 19; StataCorp LLC, College Station, TX, USA). Continuous variables were assessed for normality and analyzed using Welch’s *t*-test or ANOVA for parametric data, and Wilcoxon rank-sum or Kruskal–Wallis tests for non-parametric data, as appropriate. Categorical variables were compared using the Pearson chi-squared test. Mortality was further summarized with effect measures including absolute risk reduction (ARR), relative risk reduction (RRR), risk ratio (RR), AND odds ratio (OR). Monthly ICU mortality trends were calculated and visualized to examine longitudinal effects. Multivariable logistic and linear regression models were constructed to adjust for baseline differences and evaluate independent associations between the intervention and APACHE-IV scores and mortality. A *p*-value < 0.05 was considered statistically significant.

## 5. Results

### 5.1. Demographics

The majority of patients were White (63.3%) and male (54.0%). Black patients comprised 27.1% of the cohort, followed by Asian (5.4%) and Other/Unknown (4.0%). There were no statistically significant differences in gender (*p* = 0.965) or race (*p* = 0.104) between the PRE and POST groups. There was no difference in comorbidities between PRE and POST patients (Table 1).

### 5.2. Illness Severity Scores

**Figure 2:**
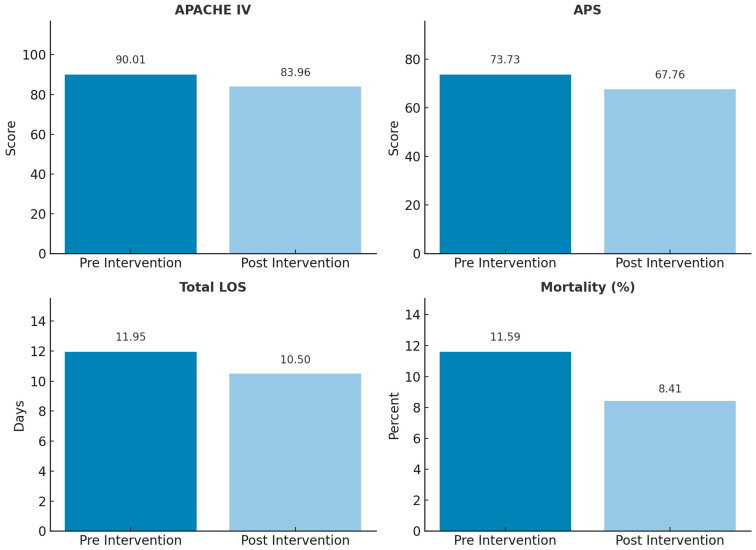
Comparison of pre- and post-intervention clinical metrics.

Both APACHE IV and Acute Physiology Score (APS) were significantly lower in the post-intervention group, indicating a reduction in illness severity among patients admitted to the ICU. The mean APACHE IV score decreased from 90.01 ± 33.12 in the pre-intervention group to 83.96 ± 30.23 post-intervention, corresponding to a mean difference of −6.05 (95% CI: 2.30 to 9.79) (*p* = 0.0016). (Figure 2, Table 2). Similarly, the APS declined from 73.73 ± 30.56 pre-intervention to 67.76 ± 28.41 post-intervention, a difference of −5.97 (95% CI: 2.48 to 9.46) *p* = 0.0008. These findings suggest a clinically and statistically meaningful reduction in illness severity at the time of ICU admission following the intervention.

Daytime CERT activations in the post-intervention period showed significant reductions in APACHE IV (89.15 ± 30.04 vs. 80.34 ± 29.17; *p* = 0.0006) and APS (72.99 ± 27.53 vs. 64.47 ± 27.67; *p* = 0.0003). Nighttime activations showed no significant changes (APACHE IV: 91.08 ± 31.30 vs. 88.15 ± 29.57, *p* = 0.3218; APS: 74.64 ± 28.76 vs. 71.56 ± 28.21, *p* = 0.2634). Across all four day/night × pre/post groups, both APACHE IV and APS scores demonstrated highly significant variation by ANOVA (APACHE IV *p* = 0.0006; APS *p* = 0.0004), indicating consistent differences in illness severity between groups (Table 2 and Table 3).

In survivors, daytime APACHE IV scores decreased significantly following the intervention (85.33 ± 29.19; median 87 [IQR 66–110] pre-intervention vs. 78.34 ± 28.66; post-intervention; *t*-test *p* = 0.0027. Nighttime APACHE IV scores showed a nonsignificant decrease (87.67 ± 31.21) pre-intervention vs. 85.15 ± 28.70 (post-intervention).

For APS, daytime values also declined significantly (69.40 ± 26.31) pre-intervention vs. 62.76 ± 27.22 post-intervention *p* = 0.0020, whereas nighttime APS changes were nonsignificant (71.46 ± 28.95 vs. 68.73 ± 26.69 *p* = 0.2634). Overall comparisons were significant on ANOVA (APACHE IV *p* = 0.0043; APS *p* = 0.0038). These findings indicate that physiologic severity at ICU admission improved significantly during daytime hours post-intervention, while nighttime values remained unchanged.

In univariate linear regression models, admission during the post-intervention period was associated with significantly lower APACHE IV scores compared to the pre-intervention period (β = −6.05, 95% CI −9.85 to −2.25, *p* = 0.002), suggesting reduced illness severity at ICU admission, potentially due to earlier recognition of physiological deterioration. Increasing age was independently associated with higher APACHE IV scores (β = 0.70 per year, 95% CI 0.58–0.82, *p* < 0.001). Gender was not significantly associated with APACHE IV score (β = −1.08 for males vs. females, 95% CI −4.85 to 2.70, *p* = 0.576). Race was also not significantly associated; compared with White patients, Black patients demonstrated a nonsignificant increase (β = 1.87, *p* = 0.396), and patients categorized as Other/Unknown had a marginal but nonsignificant increase (β = 5.16, *p* = 0.129) (Table 4).

In a multivariable linear regression evaluating predictors of illness severity (APACHE IV score), patients in the post-intervention group had significantly lower APACHE scores compared to the pre-intervention group (β = −5.71, *p* = 0.002). Older age was associated with higher APACHE scores (β = 0.70 per year, *p* < 0.001), while gender and race were not significant predictors. The model explained approximately 11.5% of the variance in APACHE scores (R^2^ = 0.115, n = 1115).

### 5.3. ICU Mortality

ICU mortality declined following the intervention, from 11.59% (95% CI: 9.28–13.90%) in the pre-intervention cohort to 8.41% (95% CI: 5.89–10.93%) post-intervention.

Daytime survival was 91.97% post-intervention vs. 88.98% pre-intervention. Nighttime survival was 91.16% post-intervention vs. 87.71% pre-intervention; however, it was not statistically significant. [*p* = 0.341].

In univariate logistic regression analyses, there was a trend toward higher odds of ICU mortality in the pre-intervention period compared to the post-intervention period (OR 1.43; 95% CI: 0.95–2.14; *p* = 0.085), though this did not reach statistical significance. Higher APACHE IV scores were strongly associated with increased odds of ICU mortality (OR 1.03 per point increase; 95% CI: 1.02–1.03; *p* < 0.001). Age was marginally associated with ICU mortality (OR 1.01 per year; 95% CI: 1.00–1.03; *p* = 0.061). Gender was not significantly associated with ICU mortality (Male vs. Female: OR 1.03; 95% CI: 0.70–1.52; *p* = 0.862). Similarly, race was not significantly associated with ICU mortality, with Black (OR 1.15; 95% CI: 0.74–1.78; *p* = 0.538) and Other/Unknown (OR 1.50; 95% CI: 0.81–2.78; *p* = 0.198) groups showing non-significant trends compared to White patients (Table 4 and Table 5).

ICU mortality rates and treatment effect estimates before and after intervention. The first column presents unadjusted estimates derived from 2 × 2 contingency table analysis using Pearson’s chi-squared test. The second column shows values adjusted for age using logistic regression.

In a multivariable logistic regression model adjusting for APACHE IV score, age, gender, and race, there was no significant association between the intervention period and ICU mortality. Patients in the pre-intervention period had 18% higher odds of ICU mortality compared to the post-intervention period, though this was not statistically significant (OR 1.18; 95% CI: 0.77–1.81; *p* = 0.458). APACHE IV score remained a strong independent predictor of ICU mortality (OR 1.03 per point; 95% CI: 1.02–1.03; *p* < 0.001). Age, gender, and race were not significantly associated with ICU mortality. Specifically, male gender (OR 1.08; 95% CI: 0.71–1.62; *p* = 0.725), Black race (OR 1.15; 95% CI: 0.72–1.84; *p* = 0.548), and Other/Unknown race (OR 1.31; 95% CI: 0.68–2.55; *p* = 0.418) showed no statistically significant differences compared to their reference groups.

The results from the chi2 analysis corresponded to an absolute risk reduction (ARR) of 3.18% (95% CI: −0.52% to 6.87%), a relative risk reduction (RRR) of approximately 27% (95% CI: −10% to 52%), a relative risk (RR) of 0.73 (95% CI: 0.48–1.10), and an odds ratio (OR) of 0.70 (95% CI: 0.45–1.07). The number needed to treat (NNT) was 32 (95% CI: 15 to ∞), indicating that treating 32 patients under the post-intervention protocol would prevent one additional ICU death. Statistical comparison yielded a *p*-value of 0.084, suggesting a trend toward improved survival.

After adjusting for age, the mortality difference remained clinically meaningful. The adjusted pre- and post-intervention mortality rates were 11.55% and 8.44%, respectively, yielding an ARR of 3.12%, RR of 0.731, RRR of 26.9%, and OR of 0.705. The NNT was 32, and the PAF was 26.9%, indicating that more than a quarter of ICU deaths could potentially be prevented through the intervention. Applying the adjusted post-intervention mortality rate to the pre-intervention cohort similarly suggests that approximately 21 deaths may have been averted, corresponding to a 26.9% reduction in ICU mortality. When comparing the time of CERT activation, survival was highest in Post-Day (91.97%) and lowest in Pre-Night (87.71%), with no significant difference across all groups (*p* = 0.341).

### 5.4. Pre-ICU Length of Stay

Overall, night activations in the total cohort, pre-ICU LOS decreased with a median of 4.10 [1.60–9.70] pre intervention and 3.60 days [1.35–8.90] post intervention (*p* = 0.1866).

In the total cohort, daytime CERT activations showed pre-ICU LOS pre- vs. post-intervention 3.68 [1.09–8.48] vs. 3.80 [1.32–8.39] (*p* = 0.8169). At night, mean pre-ICU LOS decreased with medians falling from 4.20 [1.40–9.80] to 2.90 days [1.20–7.60] (*p* = 0.0410).

In survivors, overall pre-ICU LOS declined with medians of 3.90 [1.50–9.10] and 3.20 days [1.30–8.40] *p* = 0.1821, respectively. Among survivors, daytime medians shifted from 3.68 [1.09–8.48] vs. 3.80 [1.32–8.39] (*p* = 0.8183). At night, it decreased from 4.00 [IQR 1.12–8.67] to 2.54 days [IQR 0.87–7.14] (*p* = 0.0384).

Across the four day/night × pre/post groups, pre-ICU LOS differed significantly by Kruskal–Wallis testing in the total cohort (*p* = 0.0363) and in survivors (*p* = 0.0501).

### 5.5. ICU Length of Stay

The median ICU LOS declined from 5.44 days (IQR: 2.68–11.16) to 4.91 days (IQR: 2.37–9.14) however, it did not reach statistical significance (*p* = 0.1313). Daytime ICU LOS was 5.31 [2.68–10.93] vs. 4.93 [2.32–9.00] (*p* = 0.2029). Nighttime ICU LOS: 5.73 [2.67–11.31] 4.71 [2.60–9.78] (*p* = 0.3785).

The median ICU LOS amongst survivors dropped from 5.85 days (IQR: 3.00–11.58) to 5.07 days (IQR: 2.59–9.22), reaching borderline significance (*p* = 0.0565). Daytime ICU amongst survivors decreased from 5.45 [3.01–11.28] to 5.09 [2.36–9.09] (*p* = 0.1467). Nighttime LOS decreased from 6.20 [3.23–11.19] to 5.24 [2.53–9.60], (*p* = 0.1956).

These results suggest that among patients who were discharged, ICU stays may have been shortened post-intervention, aligning with early recognition of physiological deterioration, improved care coordination, or discharge readiness.

### 5.6. Total Length of Stay (LOS)

The median total LOS decreased from 11.96 days (IQR: 6.41–20.28) to 10.58 days (IQR: 6.09–18.46), approaching statistical significance (*p* = 0.0515).

Daytime median total LOS decreased from 11.86 [6.74–19.14] to 11.36 [6.23–18.82] (*p* = 0.3856). Nighttime median total LOS decreased from 12.12 [6.30–21.01] 10.24 [6.01–17.90], approaching statistical significance (*p* = 0.0559).

Total LOS medians amongst survivors were 11.95 days (IQR: 6.57–20.40) and 10.50 days (IQR: 6.01–18.17), *p* = 0.0278, indicating a statistically significant reduction.

Daytime total LOS medians amongst survivors decreased from 11.89 [7.50–19.90] to 11.07 [6.62–18.63] (*p* = 0.2979), while nighttime median LOS decreased from 11.82 [6.99–20.23] to 10.00 [5.78–17.16] (*p* = 0.0369).

## 6. Discussion

Several studies have explored the role of remote patient monitoring (RPM) and the timing of clinical emergency response team (CERT) or rapid response team (RRT) activation in influencing patient outcomes. Earlier evaluations of continuous and automated vital sign (VS) monitoring were often limited by small sample sizes and methodological heterogeneity, yielding inconclusive results (Table 6) [6]. Our findings contribute to this evolving evidence base by demonstrating that RPM-enabled surveillance in conjunction with hospital escalation protocols may reduce illness severity at ICU transfer and improve in-hospital mortality among patients experiencing clinical deterioration.

Nighttime monitoring gaps have been identified as a critical vulnerability in acute care. Studies have shown that patients are less frequently assessed at night, contributing to delayed detection of deterioration [13]. Delays in escalation of care have been directly associated with higher short-term mortality; for example, latency in decompensation to RRT activation has been linked to increased 7-day mortality [4]. Fernando et al. further demonstrated that nighttime RRT activations were associated with significantly higher mortality than daytime activations, underscoring the importance of timely escalation. Our results may suggest reduced mortality among patients monitored with RPM and requiring CERT activation, as well as lower APACHE-IV scores at ICU admission and decreased pre-ICU LOS. We observed a reduction in the median ICU length of stay (LOS) among survivors. In addition, total hospital median LOS was reduced in survivors also decreased. There was also a trend toward shorter median pre-ICU LOS in survivors. Notably, significant reductions were seen in median pre-ICU LOS and total LOS when comparing pre-intervention nights to post-intervention nights. These findings suggest earlier recognition of physiological deterioration and more timely intervention during nighttime hours following the intervention, potentially mitigating illness progression and reducing overall hospitalization burden in survivors. Change in mortality did not differ from day to night, suggesting similar effectiveness across shift times.

These results support the interpretation that a multi-pronged approach to patients likely to deteriorate that is centered around RPM integration may reduce illness severity at ICU transfer without prolonging hospital stay. This aligns with prior literature on the role of continuous monitoring in facilitating earlier intervention and improving clinical trajectories. The decreased illness severity scores in daytime compared to nighttime may be attributable to the presence of increased resources and personnel during the day and thus, earlier detection and response to physiological deterioration.

Several related interventions have reported similar outcomes. Kadar et al. found that telemonitoring critically ill emergency department patients awaiting ICU transfer reduced in-hospital mortality and increased downgrades to non-ICU care [10]. Watanabe et al. reported improved ICU and hospital mortality, shorter LOS, and reduced EMR workload following Tele-ICU implementation [11]. Eddahchouri et al. observed decreases in unplanned ICU transfers and RRT activations with wearable wireless vs. monitors [12]. Rowland et al. demonstrated significantly lower odds of ICU transfer or death with continuous monitoring versus intermittent vs. manual checks (OR 0.36), and higher odds of adverse outcomes in patients with intermittent monitoring (OR 2.79, 95% CI 1.89–4.25). A 2025 propensity-matched study also found that continuous wireless monitoring reduced adverse composite outcomes [20].

Balshi et al. found that implementation of teleRRT decreased CPR events and mortality while increasing RRT activation rates, reinforcing the benefit of structured, real-time escalation pathways [13]. Brown et al. (2014) similarly reported a reduction in ICU days, code-blue events, and length of stay with continuous monitoring, despite no change in ICU transfer rates [5]. A cost analysis study also estimated significant financial savings associated with continuous vs. monitoring by reducing complications and improving outcomes [21].

Our findings suggest that RPM integration into hospital workflows, alongside escalation protocols and AI-assisted alerts, may improve outcomes in patients requiring transfer to the ICU by detecting physiological deterioration earlier, allowing for timely management and improved triage efficiency. Although it is difficult to isolate the effect of RPM from other elements of the intervention bundle, the reduction in APACHE-IV scores, APS, and mortality suggests meaningful clinical benefit. Our results show that after adjusting for confounding factors, APACHE scores and mortality were significantly lower in the post-intervention intervention, further validating the intervention’s role in earlier detection of physiological deterioration.

### 6.1. Strengths

This study has several strengths. It employed a real-world, multifaceted implementation strategy that included wearable technology, risk stratification tools, automated alerting, and structured CERT responses. By focusing on ICU transfers, the study selectively identified patients experiencing clinically significant deterioration. The use of a commercially available RPM system enhances replicability in other institutions. The observed reduction in APACHE-IV scores and in-hospital mortality suggests that earlier detection translated into decreased physiological deterioration. Finally, the large sample size and diverse patient population improve the robustness and external validity of findings.

### 6.2. Limitations

This study has several limitations. It was a retrospective, non-randomized, single-center analysis, which may limit generalizability. Improvements observed post-intervention may be influenced by broader institutional trends or unmeasured confounders. The assignment of ICU transfers relies on clinician judgment and bed availability, introducing potential selection bias. Additionally, RPM was implemented as part of a broader suite of interventions—including alert validation, automated escalation, and team-based responses—making it difficult to isolate the effect of RPM alone.

Moreover, the rollout of RPM occurred in a phased manner across hospital units, resulting in different start dates for implementation. As a result, the pre- and post-intervention periods varied by unit, and the overall number of months included in each group was not equivalent. While this approach reflects real-world operational constraints, it introduces potential temporal confounding and limits the ability to control for time-related changes in care processes. However, the heterogeneity of the population enhances the relevance and applicability of the findings across diverse clinical settings.

### 6.3. Clinical Implications

Our results demonstrate that RPM integration may reduce illness severity at the time of ICU admission and lower in-hospital mortality among patients requiring CERT activation. In this evaluation, ICU mortality decreased from 11.5% to 7.8% following implementation of RPM and associated interventions, corresponding to an absolute risk reduction (ARR) of 3.7% and an estimated 25 deaths prevented in the post-RPM cohort based on the unadjusted analysis.

The mean APACHE IV score decreased by approximately six points post-intervention, suggesting that patients were admitted to the ICU at an earlier stage of physiological decline. This likely reflects improved recognition and triage of clinical deterioration rather than a shift in baseline patient risk. These findings support the role of continuous physiological monitoring as part of a scalable, system-wide strategy to improve patient safety and operational efficiency in large hospitals.

In addition to improved clinical outcomes, the observed reductions in pre-ICU, ICU, and total hospital length of stay may have important implications for healthcare system efficiency. Shorter LOS can decrease overall healthcare costs, reduce staff workload and ICU burden, increase bed availability, and alleviate the labor burden on ward staff. These operational benefits may be especially relevant in high-acuity environments where staffing and bed capacity are constrained. Together, these findings possibly support the integration of continuous monitoring technologies like RPM into routine hospital workflows as part of a scalable strategy to potentially enhance both patient outcomes and system-wide performance.

### 6.4. Future Directions

The rollout of RPM at our institution involved iterative adjustments, including alert validation protocols, structured notification systems, and clear deployment criteria for in-person CERT evaluation. While our results are encouraging, further prospective, multi-center studies are needed to determine the independent impact of each component and to optimize clinical workflows. Future research should also assess long-term outcomes, patient-centered metrics, and cost-effectiveness across diverse care settings. Furthermore, future research should explore methods of leveraging frequent vital monitoring at night amidst decreased resources compared to daytime.

## 7. Conclusions

The integration of RPM, supported by structured escalation protocols and real-time clinical decision support, was associated with statistically significant reductions in APACHE-IV scores and in-hospital mortality among ICU-transferred patients following CERT activation. While causal attribution is limited by study design and a multitude of confounding variables that may be difficult to identify, the findings suggest that a comprehensive RPM strategy may enable earlier recognition of physiological deterioration and improve outcomes in hospitalized patients. Continued refinement and evaluation of RPM-enabled care models will be essential to optimizing patient safety and hospital performance. Given our results, further exploration of RPM in escalation protocols in various hospital settings is warranted.

## Figures and Tables

**Table 1:** Demographics and Comorbidities by Intervention Group.

Variable	Post (n = 464)	Pre (n = 656)	Total (N = 1120)	Chi^2^ *p*-Value
Gender				
Female	213 (45.9%)	302 (46.0%)	515	0.965
Male	251 (54.1%)	354 (54.0%)	605	
Race				
White	295 (63.6%)	414 (63.1%)	White	0.104
Black	136 (29.3%)	168 (25.6%)	Black	
Asian	18 (3.9%)	43 (6.6%)	Asian	
Other/Unknown ^‡^	15 (3.2%)	30 (4.6%)	Other/Unknown ^‡^	
Comorbidities				
CHF	214 (40.07%)	320 (59.93%)	534	0.380
COPD	68 (45.33%)	82 (54.67%)	150	0.297
DM	203 (40.85%)	294 (59.15%)	497	0.723
Hypertension	352 (41.56%)	495 (58.44%)	847	0.876
Liver Disease	112 (43.75%)	144 (56.25%)	256	0.391
CHF	214 (40.07%)	320 (59.93%)	534	0.380

^‡^ Includes Unknown, Native American, Pacific Islander, and Other.

**Table 2:** Pre vs. Post-Intervention Outcomes.

	Mean ± SD (Pre) N: 656	Mean ± SD (Post) N: 464	Mean Difference (95% CI)	*p*-Value
Total Population				
APACHE IV	90.01 ± 33.12	83.96 ± 30.23	−6.05 (2.30 to 9.79)	0.0016
APS	73.73 ± 30.56	67.76 ± 28.41	−5.97 (2.48 to 9.46)	0.0008
	Median IQR	Median IQR		
Pre-ICU LOS	4.1 (1.6–9.7)	3.6 (1.35–8.9)		0.1866
ICU LOS	5.44 (2.68–11.16)	4.91 (2.37–9.14)		0.1313
Total LOS	11.96 (6.41–20.28)	10.58 (6.09–18.46)		0.0515
Survivors				
Pre-ICU LOS	3.9 (1.5–9.1)	3.2 (1.3–8.4)		0.1821
ICU LOS	5.85 (3.00–11.58)	5.07 (2.59–9.22)		0.0565
Total LOS	11.95 (6.57–20.40)	10.50 (6.01–18.17)		0.0278

**Table 3:** LOS and Illness Severity by Time of Day and Intervention Status.

Variable	Time	Pre (n)	Post (n)	Pre Mean SD	Post Mean SD	*p*-Value
Total Population						
APACHE IV	Day	363	249	89.15 ± 32.06	80.34 ± 29.58	0.0006
	Night	293	215	91.08 ± 34.40	88.15 ± 30.50	0.3218
APS	Day	363	249	72.99 ± 29.34	64.47 ± 27.90	0.0003
	Night	293	215	74.64 ± 32.04	71.56 ± 28.58	0.2634
				Pre Median [IQR]	Post Median [IQR]	
ICU LOS	Day	363	249	5.31 [2.68–10.93]	4.93 [2.32–9.00]	0.2029
	Night	293	215	5.73 [2.67–11.31]	4.71 [2.60–9.78]	0.3785
Pre-ICU LOS	Day	363	249	4.10 [1.60–9.70]	4.80 [1.70–10.20]	0.8169
	Night	293	215	4.20 [1.40–9.80]	2.90 [1.20–7.60]	0.0410
Total LOS	Day	363	249	11.86 [6.74–19.14]	11.36 [6.23–18.82]	0.3856
	Night	293	215	12.12 [6.30–21.01]	10.24 [6.01–17.90]	0.0559
Survivors						
	Time	Pre (n)	Post (n)	Pre Median [IQR]	Post Median [IQR]	*p*-value
APACHE IV	Day	323	229	85.33 ± 29.19	78.34 ± 28.66	0.0369
	Night	257	196	87.67 ± 31.21	85.15 ± 28.70	0.3793
APS	Day	323	229	69.40 ± 26.31	62.76 ± 27.22	0.0041
	Night	257	196	71.46 ± 28.95	68.73 ± 26.69	0.3038
ICU LOS	Day	323	229	5.45 [3.01–11.28]	5.09 [2.36–9.09]	
	Night	257	196	6.20 [3.23–11.19]	5.24 [2.53–9.60]	0.1467
Pre-ICU LOS	Day	323	229	3.68 [1.09–8.48]	3.80 [1.32–8.39]	0.1956
	Night	257	196	4.00 [1.12–8.67]	2.54 [0.87–7.14]	0.8183
Total LOS	Day	323	229	11.89 [7.50–19.90]	11.07 [6.62–18.63]	0.0384
	Night	257	196	11.82 [6.99–20.23]	10.00 [5.78–17.16]	0.2979

**Table 4:** ICU Mortality and Illness Severity.

Variable	ICU Mortality OR (95% CI)—Univariable	*p*-Value	ICU Mortality OR (95% CI)—Multivariable	*p*-Value	APACHE IV β (95% CI)—Univariable	*p*-Value	APACHE IV β (95% CI)—Multivariable	*p*-Value
Pre vs. Post	1.43 (0.95–2.14)	0.085	1.18 (0.77–1.81)	0.458	6.05 (2.25 to 9.85)	0.002	5.71 (2.09 to 9.33)	0.002
APACHE IV Score	1.03 (1.02–1.03)	<0.001	1.03 (1.02–1.03)	<0.001	—	—	—	—
Age	1.01 (1.00–1.03)	0.061	1.00 (0.98–1.01)	0.578	0.70 (0.58 to 0.82)	<0.001	0.70 (0.58 to 0.82)	<0.001
Gender (Male)	1.03 (0.70–1.52)	0.862	1.08 (0.71–1.62)	0.725	−1.08 (−4.85 to 2.70)	0.576	−0.79 (–4.36 to 2.78)	0.664
Race								
Black vs. White	1.15 (0.74–1.78)	0.538	1.15 (0.72–1.84)	0.548	1.87 (−2.45 to 6.18)	0.396	2.11 (−1.96 to 6.19)	0.309
Other vs. White	1.50 (0.81–2.78)	0.198	1.31 (0.68–2.55)	0.418	5.16 (−1.51 to 11.82)	0.129	6.32 (0.02 to 12.62)	0.049

**Table 5:** ICU Mortality.

ICU Mortality	Estimate (95% CI)	Adjusted for Age (95% CI)
Metric	Estimate (95% CI)	Adjusted for Age (95% CI)
Pre-intervention mortality	11.59% (9.28–13.90)	11.55% (9.11–13.99)
Post-intervention mortality	8.41% (5.89–10.93)	8.44% (5.91–10.97)
Absolute Risk Reduction (ARR)	3.18% (−0.52–6.87)	3.12% (−0.40–6.63)
Relative Risk (RR)	0.73 (0.48–1.10)	0.731 (0.530–1.010)
Relative Risk Reduction (RRR)	27% (−10–52)	26.9%
Odds Ratio (OR)	0.70 (0.45–1.07)	0.705 (0.469–1.058)
Number Needed to Treat (NNT)	32	32
*p*-value	0.0841	0.082
Estimated deaths prevented	25	21

**Table 6:** Summary of Studies.

Author(s)	Design/Setting	RPM Modality	CERT/RRT Timing Reported	Key Outcomes
Brown et al., (2014) [12]	Before-and-after study in a medical–surgical unit	Continuous vital sign monitoring	-	↓ ICU days reduced (120.1 → 63.5/1000 pts; *p* = 0.04) ↓ Code-blue events (6.3 → 0.9/1000; *p* = 0.02) ↓ Hospital LOS (4.0 → 3.6 days; *p* < 0.05) ICU transfers unchanged
Fernando et al., 2018 [13]	Multicenter RRT study (nighttime activations)	Not RPM, but related to RRT activation timing	Nighttime vs. daytime RRT outcomes	↑ Mortality with nighttime RRT activation
Downey et al., 2018 [15]	Pilot cluster RCT, surgical wards	Wireless chest patch, 2 min interval	-	↓ Time to antibiotics (*p* = 0.04) ↓ LOS, ↓ 30-day readmissions (non-significant due to THE small sample size)
Kadar et al., 2019 [16]	ED telemonitoring of critically ill patients awaiting ICU transfer	Telemonitoring via remote intensivist oversight (eICU)	Indirect—surrogate for early CERT/RRT activation	↓ In-hospital mortality (5.4% vs. 20.0%); adjusted OR 0.20
Eddahchouri et al., 2022 [17]	Before-and-after ward study	Wireless vital sign monitors	Yes: RRT and ICU transfer rates reported	↓ ICU transfers (3.4% → 2.3%); ↓ RRT activations
Balshi et al., 2022 [18]	Before-and-after with Tele-RRT	Patient safety network with remote alerts	Tele-RRT activation tracked	↓ CPR events; increased RRT activations. ↓ Hospital mortality
Watanabe et al., 2023 [19]	Retrospective study of tele-ICU implementation across Japanese ICUs for ED patients awaiting ICU transfer	Tele-ICU monitoring with remote intensivist consultation	-	↓ ICU mortality (8.5% → 3.8%) ↓ Hospital mortality (12.4% → 7.7%) ↓ LOS ↓ Physician frequency to EMR ↓ Predicted mortality (based on severity, measured by APACHE-IV scores) found reductions, especially significant in medium in higher-risk patients
Rowland 2025 et al., [20]	Propensity-matched ward monitoring study	Continuous vs. intermittent monitoring	-	Intermittent monitoring associated with significantly higher odds of ICU transfer or death compared to continuous monitoring (OR 2.79; 95% CI 1.89–4.25; *p* < 0.001).

RRT: rapid response team; RPM: remote patient monitoring; LOS: length of stay; EMR: electronic medical records. ↓: decrease; →: to; ↑: increase.

## Data Availability

The datasets presented in this article are not readily available due to institutional restrictions.

## Supplementary Materials

The following supporting information can be downloaded at https://www.mdpi.com/article/10.3390/jcm14207434/s1. Figure S1: Biobutton; Table S1: Remote Patient Monitoring Launch Dates by Unit.

## Abbreviations

VS	Vital signs
RPM	Remote patient monitoring
RRS	Rapid response system
CERT	Clinical emergency response team
LOS	Length of stay
ICU	Intensive care unit
vICU	Virtual intensive care unit
