# Supplementary material for: Implementation of Remote Patient Monitoring and Earlier CERT Activation: Effects on ICU Transfer and Mortality
Source: J Clin Med. 2025 Oct 21;14(20):7434. doi: 10.3390/jcm14207434 (PMC12565538; doi:10.3390/jcm14207434)

Supplementary Table S1. Remote Patient Monitoring Launch Dates by Unit

Row Labels	Bio Go-live date
Medical Unit 1	11/6/2023
Medical Unit 2	9/25/2023
Medical Unit 3	9/25/2023
Medical Unit 4	9/25/2023
Medical Unit 5	9/25/2023
Medical Unit 6	9/25/2023
Medical Unit 7	9/25/2023
Medical Unit 8	11/6/2023
Medical Unit 9	11/6/2023
Medical Unit 18	2/28/2024
Medical Unit 19	11/6/2023
Medical Unit 20	4/24/2023
Medical Unit 21	4/24/2023
Medical Unit 22	11/6/2023
Medical Unit 23	11/6/2023
Medical Unit 24	11/6/2023
Medical Unit 25	11/6/2023
Surgical Unit 1	9/25/2023
Surgical Unit 2	9/25/2023
Surgical Unit 3	4/24/2023

Supplementary Figure S1. Biobutton

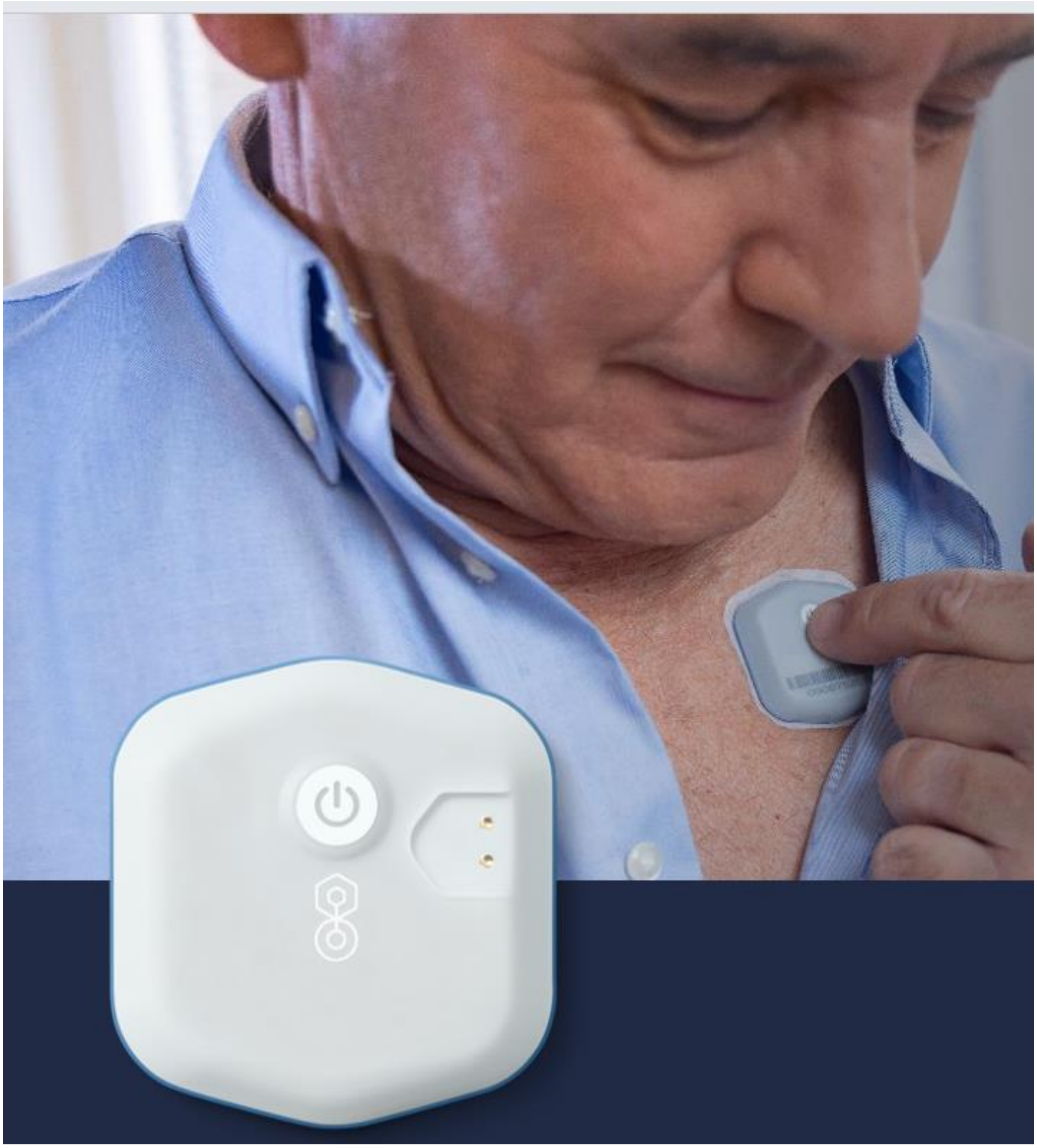

Supplement: Supplementary file 1 [file jcm-14-07434-s001.zip › jcm-3881705-supplementary.pdf]
